# Casitas B-Lineage Lymphoma RING Domain Inhibitors Protect Mice against High-Fat Diet-Induced Obesity and Insulin Resistance

**DOI:** 10.1371/journal.pone.0135916

**Published:** 2015-08-21

**Authors:** Min Wu, Lin Sun, Ziyan Yuan Pessetto, Zhihe Zang, Xingliang Xie, Ling Zhong, Qing Su, Wang Zan, Xiurong Gao, Yan Zhao, Yiyi Sun

**Affiliations:** 1 Department of Pharmacy, Chengdu Medical College. Chengdu, Sichuan Province, China; 2 West China Hospital, Sichuan University, Chengdu, Sichuan Province, China; 3 Department of Pathology and Laboratory Medicine, Univerisity of Kansas Medical Center, Kansas City, KS, United States of America; Russian Academy of Sciences, Institute for Biological Instrumentation, RUSSIAN FEDERATION

## Abstract

The casitas b-lineage lymphoma (c-Cbl) is an important adaptor protein with an intrinsic E3 ubiquitin ligase activity that interacts with E2 proteins such as UbCH7. c-Cbl plays a vital role in regulating receptor tyrosine kinase signaling. c-Cbl involves in whole-body energy homeostasis, which makes it a potential target for the treatment of type 2 diabetes and obesity. In the present study, we have designed two parental peptides and 55 modified peptides based on the structure of UbCH7 loop L1 and L2. Thirteen of the modified peptides showed increased inhibitory activity in a fluorescence polarization-based assay. In the *in vivo* proof of study principle, mice treated with peptides 10, 34, 49 and 51 were protected against high-fat diet-induced obesity and insulin resistant. These inhibitors may potentially lead to new therapeutic alternatives for obesity and type 2 diabetes.

## Introduction

The incidence of obesity and type 2 diabetes is increasing throughout the world and currently affects about 250 million people worldwide. Possible causes of this health problem are credited partially to several risk factors. History of hyperglycemia, prediabetes, and/or gestational diabetes, overweight and obesity, physical inactivity, genetics, were reported (American Diabetes Association, the diabetes advisor). Researchers described a number of genes that regulate food absorption, appetite, and increased energy expenditure in either adipose or muscle tissue over the past decade [1, 2]. The Casitas B-lineage Lymphoma protein c-Cbl is one of these genes, and it is known to regulate whole-body energy expenditure [3]. It has been recently reported that *c-Cbl* ^-/-^ mice exhibited a lean phenotype and enhanced peripheral insulin actions likely due to elevated energy expenditure [3]. After a five-week high-fat diet, *c-Cbl* ^-/-^ mice maintained hyperphagia, higher whole-body oxygen consumption, and a 3-fold greater activity compared to wild-type mice fed the same diet [2]. However, the function of the c-Cbl protein, which is responsible for the lean phenotype, remains unclear because Cbl proteins involve in both the PI3K-dependent and PI3K-independent pathways in insulin-stimulated glucose transport [2–4]. Insulin binds to its receptor and activates the receptor tyrosine kinase activity. The activation of the tyrosine kinase then leads to autophosphorylation of the receptor. Next, the Insulin Receptor Substrate (IRS) ½ is recruited and phosphorylated [5]. However, IRS-2 pathway is not involved in the glucose transporter 4 (GLUT4) trafficking [2–4]. Subsequently, other signaling molecules such as PI3K are recruited which leads to the activation of downstream signaling events. Insulin binding also leads to recruitment of Cbl and CAP proteins and leads to TC10 activation. Downstream of CAP-Cbl, signaling bifurcates into two arms; one arm is characterized by PI3K-II while the other arm may involve atypical Protein Kinase C (aPKC) and is characterized by Rac and actin remodeling. A-actin 4 (ACTN4) possibly links GLUT4 vesicles to actin filaments. Both pathways are needed to stimulate GLUT4 translocation in adipocytes [5, 6]. The data suggest that c-Cbl ubiquitin ligase activity plays an essential role for the lean phenotype, and it may be essential in regulating the expression of proteins involved in the energy expenditure control [2].

*c-Cbl* ^*A/-*^ (C379A) mice expressing mutation within the RING finger domain of c-Cbl protein were found to have very similar phenotype compared to *c-Cbl* ^-/-^ mice. *c-Cbl* ^*A/-*^ mice have reduced adipose tissues, insulin, leptin, and triglyceride levels compared to the wild-type mice [4]. They also have improved glucose tolerance compared to the wild-type mice [4]. Elevated oxygen consumption was observed. Researchers examined mice expressing a mutant c-Cbl with the PI3K binding domain ablated (*c-Cbl* ^*F/F*^) mice. These mice (*c-Cbl* ^*F/F*^) exhibited similar insulin sensitivity, body composition, and energy expenditure compared to the control wild-type mice [2–4]. Therefore, these data suggest that it is c-Cbl ubiquitin ligase activity plays an important role in the regulation of whole-body energy metabolism [4]. All of these data emphasize that c-Cbl is a promising therapeutic target for obesity and type 2 diabetes. Here we propose to generate c-Cbl RING domain inhibitors that can functionally mimic the c-Cbl RING domain mutation and knockout the c-Cbl ubiquitination function through a peptidomimetic approach. We hypothesize that c-Cbl inhibitors will protect mice against high-fat diet-induced obesity and insulin resistance.

Ubiquitination requires the sequential actions of three enzymes: (i) an activating enzyme (E1) forms a high-energy thioester bond with the ubiquitin carboxyl group of G76, thereby activating the C-terminus of ubiquitin; (ii) a conjugating enzyme (E2), which has active cysteine residues, is recruited and transiently carries the activated ubiquitin; (iii) a ligase (E3) then transfers the activated ubiquitin from the E2 to the substrate lysine residue [7]. Here we propose to design inhibitors to the E2–E3 binding interface. E2–E3 (protein-protein) interfaces are flat and expansive; there is no well-defined pocket such as with enzyme active sites. Therefore, this topology facilitates multiple signaling partners to bind to the same site [8]. Molecules derived from natural or bioactive are good candidates for screening. By enhancing hydrogen bonding, hydrophobic, or electrostatic interactions between the peptides and proteins, the potency of these peptides can increase considerably.[9].

Aqueous solubility, lipophilicity, chemical stability and metabolic stability (proteolytic and enzymatic degradation) often determine the bioavailability and biodistribution of peptide inhibitors. Because of the intrinsic physicochemical properties, peptides composed of natural amino acids are not very good candidates. These peptides are known to have low stability in plasma. They are also sensitive to proteases and can be cleared from the circulation in a few minutes [10]. Strategies to improve the activity and stability of peptide inhibitors include constraining the peptide to the bound confirmation, cyclizing the peptide, biosterically replacing the peptide bonds, changing the stereochemistry of amino acids (D conformation), and capping the peptide terminus [11–17]. Therefore, we proposed to use Fmoc-protected amino acid analogs to probe the role of each residue and to increase the activity.

## Materials and Methods

### Peptides and proteins

Peptides and peptide mimetics were purchased from MitenChem (China) with a purity ≥ 98%. Concentrations values of reconstituted peptides were determined by amino acid analysis at the protein core facility (Chengdu Medical College). The recombinant Cbl protein was produced by the Chengdu Medical College Protein Core Facility and was described previously [9]. The c-Cbl (RING) protein, including the linker and RING domain (codon 358–457), was tagged with Glutathione S-Transferase (GST) and purified with GSH-Sepharose (Shanghai Biopharma) [9].

### Fluorescence polarization (FP) assay

The FP assay was developed and validated previously [9]. Briefly, fluorescein-labeled probe (FITC-βA-PFKPP-NH_2_, 100 nmol/L) and Cbl (RING) protein (3 μmol/L) were mixed and diluted to the final concentration using 0.01% Triton X-100 in PBS (pH = 7.4). Then, 19 μl of this master mix was loaded to each reaction well. The peptides or peptide mimetics were then loaded to the designated wells (1 μl, 10 μmol/L– 0.078 μmol/L, half-fold dilutions). The total fluorescence and polarization values were measured at an excitation wavelength of 485 nm and an emission wavelength of 538 nm using a ZS-2 plate reader. The data was fitted, and the ΔmP, K_d_, and K_i_ values were estimated, using SigmaPlot 11.0.

### *In vivo* studies [2–4, 18].

All experiments were approved by the institutional Animal Care and Use Committee (Chengdu Medical College, China). All experiments were carried out in 10-week-old male mice maintained on the C57BL/6 background. All the animals were kept on a 12-h light/dark cycle with free access to food and water.

### Acute toxicity

The control group (n = 10) received vehicle only. Eight groups (10 mice each) were treated with increasing doses of either peptide 1, 3, 10, 34, 2, 60, 49, and 51 up to 15 mg/kg by i.p. Number of death, sedation, spontaneous motor activity, alertness, ptosis, dyspnea, convulsion, diarrhea, urination, postural reflex, piloerection, nociception, grooming, vocalization, rearing, climbing and aggression were observed every 12 hours for 72 h. Animals were maintained for another 14 days after the initial examination. We planned to sacrifice the animals if they show severe signs of pain or distress, a body weight loss in excess of 15% of its body weight, or a deterioration of the body condition score to BC2(-) or below. However, none of the animals qualified for the mentioned the symptoms. No animals died during the whole study. At the end of the experiment, animals were euthanized by CO_2_ asphyxiation followed by cervical dislocation, the livers, spleens and kidneys were collected and formalin-fixed paraffin-embedded for H&E staining.

### Pharmacokinetic studies

Peptides (1, 3, 10, 34, 2, 60, 49, and 51) were dissolved in sterile aqueous 5% dextrose and administered to mice by i.p. injection (4 mg/kg). Blood samples were collected from the tail tip at 0, 0.5, 1, 6, 12, 18, 24 and 48 hours. Plasma samples were harvested by centrifugation and stored at -80°C until assayed. Peptide concentrations in plasma were determined by Reversed-Phase High-Performance Liquid Chromatography (RP-HPLC) with electrospray ionization mass spectrometric (EI-MS) detection. Samples were assayed with a series of 8 calibration standards of peptide in plasma at concentrations ranging from 50 to 6000 μg/L. Peptide concentrations were determined by comparing to the standards [19, 20].

### Drug treatment for *in vivo* studies

Animals (n = 90) were randomly assigned to one of the nine groups (Table 1). Animals were fed ad libitum with a high-fat diet (60% of caloric intake from fat (70% saturated fat), 20% from carbohydrates, and 20% from protein) before experiments and for another 12 weeks during the experiments. Food intake was measured manually on a daily basis. Eight groups of animals were treated with indicated peptides with a daily i.p. injection at 5 mg/kg and one group of animals were treated with vehicle.

**Table 1:** *In vivo* study experimental groups.

**Group**	High-fat diet	Cbl RING domain inhibitor
**1**	+	-
**2**	+	Peptide 1
**3**	+	Peptide 3
**4**	+	Peptide 10
**5**	+	Peptide 34
**6**	+	Peptide 2
**7**	+	Peptide 60
**8**	+	Peptide 49
**9**	+	Peptide 51

### Body composition determination

Body composition was measured using nuclear magnetic resonance (NMR) technology at Chengdu College core facility. Epididymal fat pads were excised and adipocytes were isolated. Cells were then fixed using 2% O_s_O_4_ in PBS overnight at 37°C [2].

### Metabolic assays

After receiving 12 weeks of treatment as described above, glucose tolerance tests were performed in overnight fasted mice. Each mouse received a 2 g/kg glucose through i.p. Blood samples were obtained from the tail tip at 0, 30, 60, 90, and 120 min. Insulin concentrations were measured using mouse insulin Elisa kits (ChrystalChem) using mouse standards. Glucose levels were measured using a glucometer (Roche) [2].

#### Pancreatic islet isolation and insulin secretion assay

The methods were described by Melero et al., [2]. Briefly, the islets were isolated using liberase (Roche) digestion of the pancreas while mice were under anesthesia. Then the purification with a FicoII-Paque density gradient (Amersham Bioscience) was performed. The mice islets were incubated in Krebs Ringer buffer (2.8 or 16.7 mmol/l glucose) for one hour at 37°C. Insulin levels were measured by radioimmunoassay with the mice insulin standard.

#### Indirect energy expenditure and physical activity measurement [2]

After receiving 12 weeks of treatment as described above, rates of oxygen consumption (V_O2_) was measured using a four-chamber indirect calorimeter (Langguan, China, air flow of 0.5 l/min). A total of 2-hour measurement for each animal was conducted at 15-minuate intervals under room temperature (22°C). Ambulatory activity was estimated by counting the number of photobeam breaks in a three-sensor chamber instrument (Rui Pharm, China), and the cumulative ambulatory activity counts were recorded every hour. A total of 12 h-day time and 12 h-night activity were recorded for each animal.

### Statistical analyses

Data were presented as means ± SE. Statistical analyses were performed using the Student’s *t*-test (SigmaPlot 11.0 software). Differences were considered to be statistically significant at p < 0.05.

## Results

### Peptides derived from UbCH7 L1 and L2 loop inhibit c-Cbl and the probe binding in FP assay *in vitro*

The complex of c-Cbl and UbcH7 interactions was solved as the first RING E3–E2 structure. Later, other E3–E2 structures, including E6AP-UbcH7, CNOT4-UbcH5B, and CHIP/Bbc13-Uev1a complexes, were solved by either NMR or crystallography [21, 22]. The results indicate that the c-Cbl RING interacts with similar residues on UbcH7 and UbcH5B [22]. Therefore, we propose to choose UbcH7 as our peptide template for inhibitor design.

**Fig 1:**
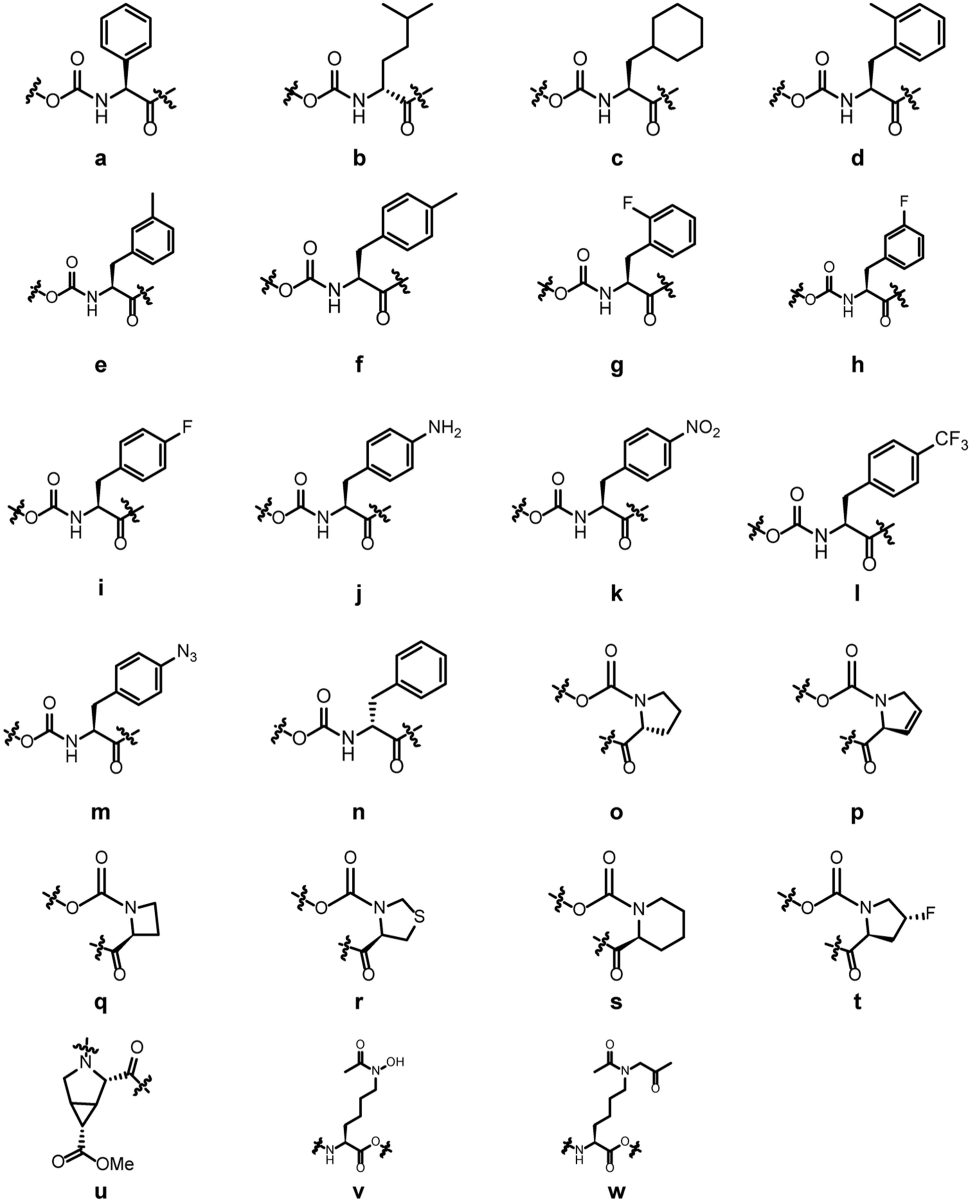
Analogs of amino acids used as surrogates to modify parental peptides.

Synthetic optimization of the peptide derived from UbCH7 Loop L1 (peptide 1), and Loop L2 (peptide 2) were summarized in Fig 1 and Tables 2 and 3. In all, 57 peptides were synthesized and the K_i_ values were examined in FP assay [9]. There are eleven peptides that showed improved activity compared to the parental peptide 1 with the K_i_ values less than 7.2 μM (Table 2 and Fig 1). There are two peptides that showed improved activity compared to the parental peptide 2 with the K_i_ values less than 4.7 μM (Table 3 and Fig 1). Peptides 10, 34, 49 and 51 which have low K_i_ values, were chosen for the following studies. We also included peptide 1 and 2 (parental peptides) and two negative peptides (peptide 3 and 60) which have high K_i_ values as controls.

**Table 2:** Sequences and inhibitory activity of Cbl RING domain inhibitors derived from UbCH7 L1.

**Peptide**	Sequence	K_i_ (μM)	Peptide	Sequence	K_i_ (μM)
**1**	Ac-PFKPP-NH_2_	7.2 ± 0.5	**21**	Ac-P(**h**)KP(**s**)-NH_2_	90.9 ± 0.7
**2**	Ac-KPATK-NH_2_	4.7 ± 1.2	**22**	Ac-P(**h**)KP(**t**)-NH_2_	31.2 ± 0.4
**3**	Ac-P(**a**)KPP-NH_2_	>500	**23**	Ac-P(**h**)KP(**u**)-NH_2_	> 250
**4**	Ac-P(**b**)KPP-NH_2_	10.5 ± 1.2	**24**	Ac-P(**h**)K(**o**)P-NH_2_	3.5 ± 0.4
**5**	Ac-P(**c**)KPP-NH_2_	125.2 ± 12.6	**25**	Ac-P(**h**)K(**p**)P-NH_2_	> 250
**6**	Ac-P(**d**)KPP-NH_2_	10.2 ± 1.5	**26**	Ac-P(**h**)K(**q**)P-NH_2_	> 250
**7**	Ac-P(**e**)KPP-NH_2_	4.5 ± 1.0	**27**	Ac-P(**h**)K(**r**)P-NH_2_	8.5 ± 10.2
**8**	Ac-P(**f**)KPP-NH_2_	5.4 ± 2.2	**28**	Ac-P(**h**)K(**s**)P-NH_2_	135.6 ± 11.2
**9**	Ac-P(**g**)KPP-NH_2_	3.5 ± 0.6	**29**	Ac-P(**h**)K(**t**)P-NH_2_	24.6 ± 5.8
**10**	Ac-P(**h**)KPP-NH_2_	1.8 ± 0.8	**30**	Ac-P(**h**)K(**u**)P-NH_2_	> 250
**11**	Ac-P(**i**)KPP-NH_2_	2.4 ± 0.5	**31**	Ac-(**o**) (**h**)KPP-NH_2_	4.5 ± 0.5
**12**	Ac-P(**j**)KPP-NH_2_	4.5 ± 0.8	**32**	Ac-(**p**) (**h**)KPP-NH_2_	> 250
**13**	Ac-P(**k**)KPP-NH_2_	4.2 ± 0.4	**33**	Ac-(**q**) (**h**)KPP-NH_2_	> 250
**14**	Ac-P(**l**)KPP-NH_2_	25.4 ± 0.7	**34**	Ac-(**r**) (**h**)KPP-NH_2_	1.5 ± 0.4
**15**	Ac-P(**m**)KPP-NH_2_	8.4 ± 0.4	**35**	Ac-(**s**) (**h**)KPP-NH_2_	122.3 ± 9.8
**16**	Ac-P(**n**)KPP-NH_2_	15.4 ± 0.4	**36**	Ac-(**t**) (**h**)KPP-NH_2_	14.5 ± 2.9
**17**	Ac-P(**h**)KP(**o**)-NH_2_	12.5 ± 0.9	**37**	Ac-(**u**) (**h**)KPP-NH_2_	> 250
**18**	Ac-P(**h**)KP(**p**)-NH_2_	> 250	**38**	Ac-(**r**) (**h**) (**v**)PP-NH_2_	21.6 ± 2.6
**19**	Ac-P(**h**)KP(**q**)-NH_2_	> 250	**39**	Ac-(**r**) (**h**) (**w**)PP-NH_2_	28.3 ± 2.8
**20**	Ac-P(**h**)KP(**r**)-NH_2_	3.7 ± 0.4			

Letters in bold in the sequence refer to the structure in Fig 1.

**Table 3:** Sequences and inhibitory activity of Cbl RING domain inhibitors derived from UbCH7 L2.

**Peptide**	Sequence	K_i_ (μM)	Peptide	Sequence	K_i_ (μM)
**2**	Ac-KPATK-NH_2_	4.7 ± 1.2	**50**	Ac-K(**s**)ATK-NH_2_	32.6 ± 10.9
**40**	Ac-(**v**)PATK-NH_2_	51.8 ± 0.8	**51**	Ac-K(**t**)ATK-NH_2_	3.8 ± 1.1
**41**	Ac-(**w**)PATK-NH_2_	96.2 ± 12.6	**52**	Ac-K(**u**)ATK-NH_2_	66.4 ± 10.8
**42**	Ac-KPAT(**v**)-NH_2_	59.8 ± 6.5	**53**	Ac-K(**r**)VTK-NH_2_	91.2 ± 6.6
**43**	Ac-KPAT(**w**)-NH_2_	72.6 ± 9.5	**54**	Ac-K(**r**)TTK-NH_2_	201.5 ± 14.4
**44**	Ac-(**v**)PAT(**w**)-NH_2_	125.6 ± 22.6	**55**	Ac-K(**r**)ITK-NH_2_	190.3 ± 19.2
**45**	Ac-(**w**)PAT(**v**)-NH_2_	195.6 ± 12.3	**56**	Ac-K(**r**)LTK-NH_2_	225.6 ± 22.5
**46**	Ac-K(**o**)ATK-NH_2_	27.3 ± 5.5	**57**	Ac-K(**t**)AAK-NH_2_	178.6 ± 10.2
**47**	Ac-K(**p**)ATK-NH_2_	44.3 ± 13.2	**58**	Ac-K(**t**)AVK-NH_2_	125.6 ± 3.2
**48**	Ac-K(**q**)ATK-NH_2_	59.6 ± 13.5	**59**	Ac-K(**t**)AIK-NH_2_	144.8 ± 18.3
**49**	Ac-K(**r**)ATK-NH_2_	2.9 ± 1.2	**60**	Ac-K(**t**)ALK-NH_2_	228.2 ± 20.2

Letters in bold in the sequence refer to the structure in Fig 1.

### Favorable safety and pharmacokinetics profiles of modified peptides

We first examined the acute toxicity and pharmacokinetic properties of peptides 1, 2, 3, 10, 34, 2, 60, 49 and 51. All animals received peptides up to 15 mg/kg by i.p. or the vehicle by i.p. and did not show vital signs of acute toxicity in 72 hours. None of the other symptoms outlined in the method section were observed during the study. There is no significant difference between all groups. None of the animals died after another 14 days following the initial examination. The H&E staining does not show any sign of injuries for the livers, spleens and the kidneys.

**Fig 2:**
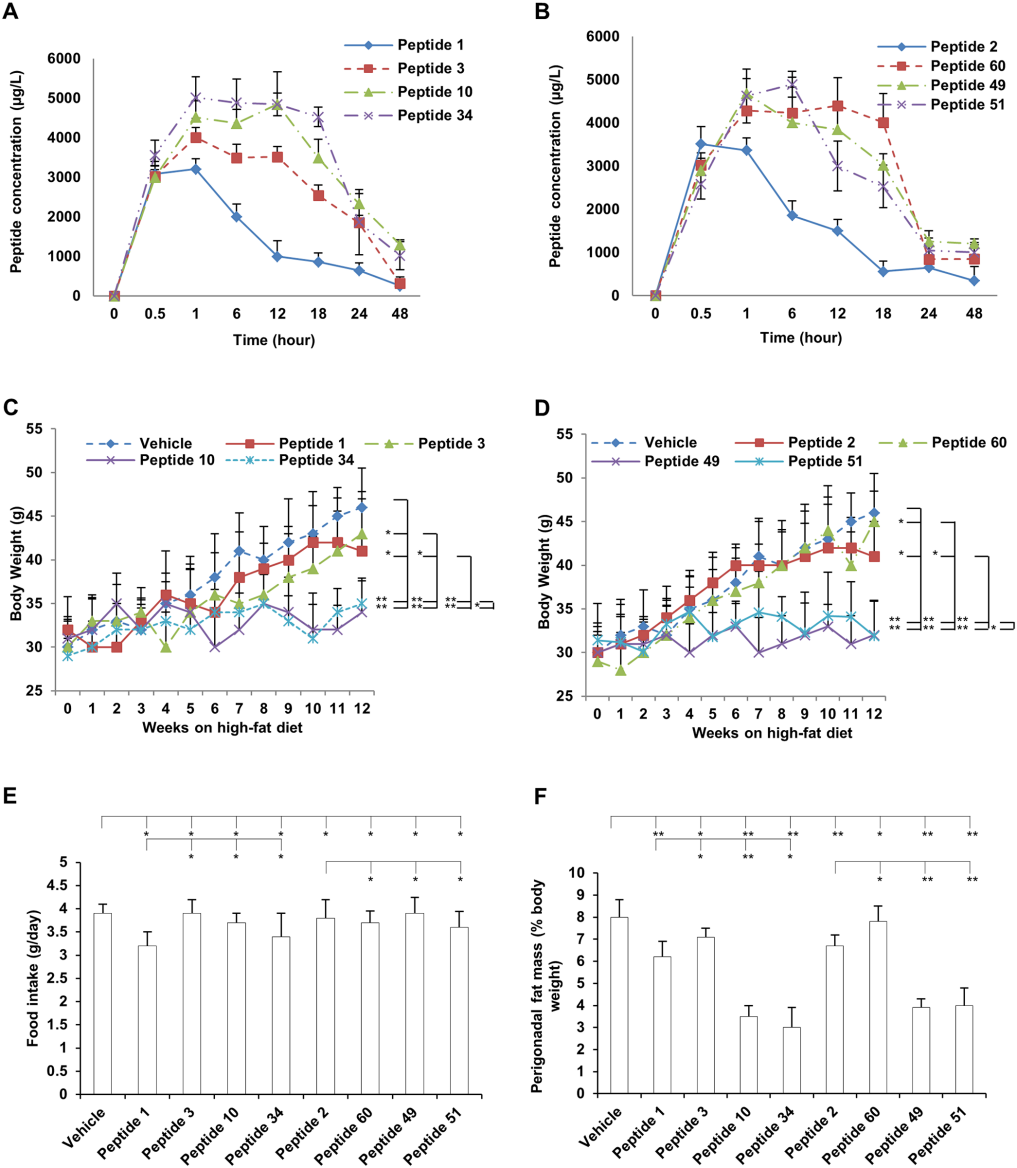
*In vivo* studies of c-Cbl inhibitors. **A-B**. Pharmacokinetic studies of parental peptides and modified peptides. Peptides were administered to mice by i.p. injection (4 mg/kg). Blood samples were collected from the tail tip at the indicated time points. Plasma samples were harvested and analyzed using RP-HPLC with EI-MS detection. **C-D**. Body weight during 12 weeks feeding with a high-fat diet. **E**. Food intake. **D**. Percentage of perigonadal fat mass. Mice were fed ad libitum with the high-fat diet before experiments and for another 12 weeks during the experiments. Mice were treated with vehicle or indicated peptides with a daily i.p. injection at 5 mg/kg. Body weight, food intake were measured manually on a daily basis. Perignonadal fat mass was measured using NMR technology. * p > 0.05. **p < 0.05.

We next examined the pharmacokinetics of these peptides. Inspection of the plasma concentration-time profiles of these peptides revealed a significant difference in the disposition of the eight peptides (Fig 2A and 2B). In particular, the maximum concentration in plasma observed in 30 min for peptide 1 and 2 were 3,085 ± 315 μg/L and 3,368 ± 285 μg/L compared to peptide 10, 34, 49 and 51 observed in 60 min were 4,521 ± 421 μg/L, 5,021 ± 524 μg/L, 4,682 ± 345 μg/L, 4,625 ± 621 μg/L. The maximum concentration in plasma observed in 60 min for the negative control peptides 3 and 60 were 4,015 ± 250 μg/L, and 4,285 ± 345. At each time point after 1 hour, the modified peptides (3, 10, 34, 49, 51, and 60) had greater plasma concentrations compared to the parental peptides 1 and 2. The negative control peptides (3 and 60) have lower plasma concentrations compared to the modified peptides (10, 34, 49 and 51) but were not statistically significant.

### Mice treated with peptides 10, 34, 49 and 51 were protected against high-fat diet-induced obesity

**Fig 3:**
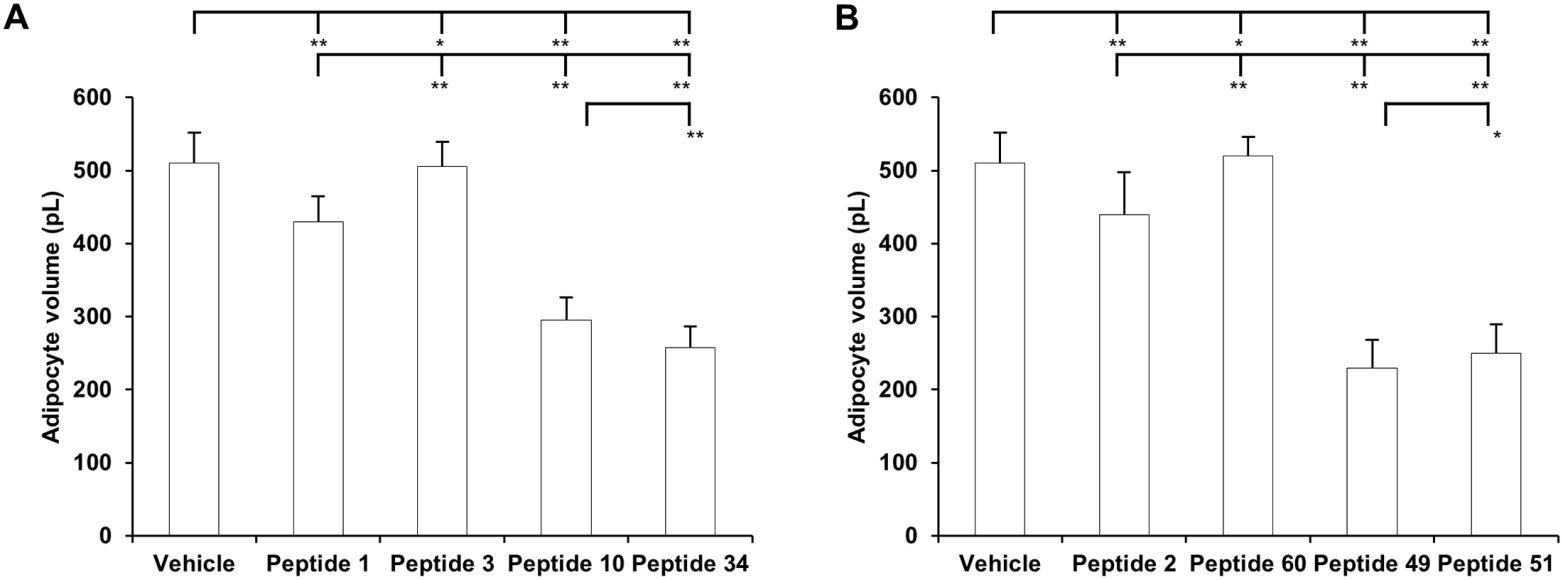
A-B. Adipocyte volume. Mice were fed ad libitum with the high-fat diet before experiments and for another 12 weeks during the experiments. Mice were treated with vehicle or indicated peptides with a daily i.p. injection at 5 mg/kg. After the experiment, isolated adipocytes were obtained from excised epididymal fat pad.

There were significant differences in body weight gain during the 12-week feeding period between the vehicle, peptide 1 and 3 treated mice groups and peptide 10 and 34 treated groups (Fig 2C). There were significant differences in body weight gain during the 12-week feeding period between the vehicle, peptide 2 and 60 treated mice groups and peptide 49 and 51 treated groups (Fig 2D). Food intake was monitored manually, and no significant difference was noticed among all groups (Fig 2E). The perigonadal fat mass (Fig 2F) showed significant differences in peptides 10, 34, 49, and 51 treated groups. The percentage of perigonadal fat mass for the vehicle, peptide 1, 3,10, 34, 2, 60, 49, and 51 treatment groups were 8 ± 0.8%, 6.2 ± 0.7%, 7.1 ± 0.4%, 3.5 ± 0.5%, 3.0 ± 0.9%, 6.7 ± 0.5%, 7.8 ± 0.7%, 3.9 ± 0.4%, and 4.0 ± 0.8%, respectively (Fig 2E). The adipocyte volume for the vehicle, peptide 1, 3,10, 34, 2, 60, 49, and 51 treatment groups were 510 ± 42 pL, 430 ± 35 pL, 505 ± 34 pL, 295 ± 31 pL, 258 ± 29 pL, 440 ± 58 pL, 520 ± 26 pL, 230 ± 38 pL, and 250 ± 40 pL, respectively (Fig 3A and 3B). The statistical significances between each group were indicated in the figures.

### Mice treated with peptides 10, 34, 49 and 51 were protected against high-fat diet-induced insulin resistance

**Fig 4:**
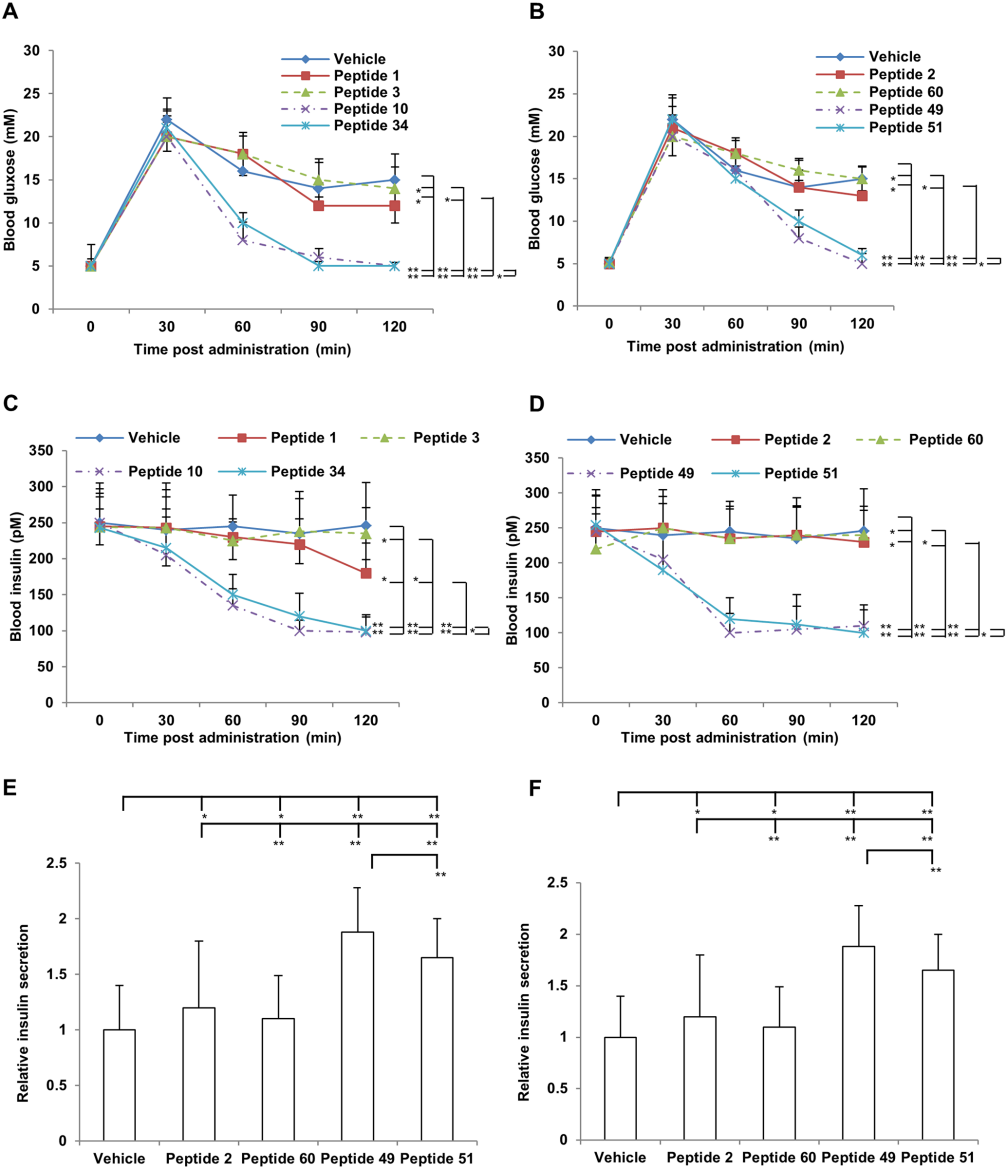
Glucose tolerance test in overnight fasted vehicle and peptides treatment groups. Mice were fed ad libitum with the high-fat diet before experiments and for another 12 weeks during the experiments. At the end of the experiment, glucose tolerance tests (2 g/kg glucose i.p.) were performed in overnight fasted mice. **A-B**. Blood glucose levels after the glucose challenge (i.p. 2 g/kg). **C-D**. Insulin secretion from pancreatic islets. **E-F**. Relative insulin secretion. Data are presented as means ± SE. ** P<0.05.

Glucose tolerance tests were performed after 12 weeks of treatment as indicated above. The vehicle group showed an impaired glucose clearance (Fig 4A and 4B). The peptides 10, 34, 49 and 51 treatment groups showed better glucose tolerance compared to the vehicle group (Fig 4A and 4B). A significant reduction in blood insulin levels were also observed in the peptides 10, 34, 49 and 51 groups compared to the vehicle group (Fig 4C and 4D). In addition, we observed that were differences between the parental peptides 1 and 3 treatment groups and vehicle groups. However, these differences were not statistically significant. In all, the peptides 10, 34, 49 and 51 treatment groups for both blood glucose levels and blood insulin levels were better than the vehicle groups and the parental peptides treatment groups. These data indicate that peptides 10, 34, 49 and 51 have a better efficacy for improving glucose tolerance.

### Mice treated with peptide 10, 34, 49 and 51 and 34 exhibit increased oxygen consumption and ambulatory activity compared to the vehicle-treated mice and mice treated with peptide 1, 3, 2 and 60

Table 4 summarized the indirect energy expenditure and physical activity measurement results. Mice treated with Cbl-RING domain inhibitors (peptide 10, 34, 49 and 51) exhibit higher energy expenditure than the mice treated with vehicle, parental peptides (peptide 1 and 2) and the negative control peptides (peptide 3 and 60). Cbl-RING domain inhibitors (peptide 10, 34, 49 and 51) treated mice exhibit higher ambulatory activity both day and night than the vehicle, parental peptides (peptide 1 and 2) and the negative control peptides (peptide 3 and 60) treated mice.

**Table 4:** Indirect energy expenditure and physical activity measurements result.

Group	Oxygen consumptionV_O2_ (ml·g^-1^·h^-1^)	Ambulatory day activity (per 12 h)	Ambulatory night activity (per 12 h)
**1 (Vehicle)**	4.15 ± 0.55	2,069 ± 705	6,289 ± 1,216
**2 (Peptide 1)**	3.95 ± 0.22	2,152 ± 865	5,941 ± 1,355
**3 (Peptide 3)**	3.78 ± 0.29	2,158 ± 763	6,058 ± 1,298
**4 (Peptide 10)**	5.92 ± 0.36	3,512 ± 923	12,896 ± 2,469
**5 (Peptide 34)**	5.88 ± 0.85	3,485 ± 864	1,5624 ± 3,216
**6 (Peptide 2)**	4.22 ± 0.42	2,342 ±1,002	6,721 ± 1,032
**7 (Peptide 60)**	4.16 ± 0.29	2,025 ± 945	5,993 ± 1,278
**8 (Peptide 49)**	6.12 ± 0.58	4,012 ±1,207	20,126 ± 2,914
**9 (Peptide 51)**	5.99 ± 0.95	4,953 ± 1,014	19,251 ± 3,058

## Discussion

Analogs (**a-w**) were used as substitutes for the natural amino acid residue to increase the activity and stability (Fig 1). Phenylalanine residue in peptide **1** plays a critical role in the c-Cbl-UbcH7 binding interaction. To explore the tolerance of extensions to the phenyl ring, analogs (**a-n**) were used as surrogates (Fig 1). Analogs (**a** and **d-f**) were used to explore the size and shape of the hydrophobic patch. Analogs **b** and **c** were used to explore the significance of the phenyl ring. Analog **n** changes the stereo-conformation of the residue. Analogs **g-m** that has fluoro or hydroxyl substitutions were chosen to explore potential hydrogen-bonds with the protein, while the methyl (**d** and **e**), cyclohexyl (**c**) and isopropyl (**b**) substitutions were also used to explore the packing interactions. Analogs (**o-u**) were used as substitutes for the proline residue to explore its role in the binding interaction. Analog **o** changes the stereochemistry of the residue. Analogs **p-t** were used to explore the role of the pyrrolidine ring. The turn induced by the proline residue is probably critical to restrict the peptide to the bound conformation. Analog **u** was used as a proline substitute because it can constrain the peptide backbone similar to a natural L-proline bond conformation. Lysine analogs (**v** and **w**) can give some extensions to the side chain and may increase the activity and stability.

The FP assay showed that modified peptides (Tables 1 and 2) have enhanced activity. The acute toxicity and pharmacokinetic assays also proved that these modified peptides are safe and tolerant to hydrolysis. The *in vivo* study showed that the modified peptides 10, 34, 49 and 51 protect mice from high-fat-diet induced obesity and insulin resistance.

In conclusion, the global incidence of obesity and diabetes is increasing at such a rate as to be characterized as an epidemic. The research conducted in this article provides several lead c-Cbl RING domain inhibitors that specifically inhibit c-Cbl-UbcH7 binding. It would be interesting to explore these mechanisms in future studies. These inhibitors may potentially lead to new therapeutic options for obesity and type 2 diabetes.
